# Exploring the biochemical landscape of bacterial medium with pyruvate as the exclusive carbon source for NMR studies

**DOI:** 10.1007/s10858-025-00462-1

**Published:** 2025-03-06

**Authors:** Çağdaş Dağ, Kerem Kahraman

**Affiliations:** 1 Nanofabrication and Nanocharacterization Center for Scientific and Technological Advanced Research (n²STAR), Koç University, İstanbul, Türkiye; 2 Koç University İşbank Center for Infectious Diseases (KUISCID), Koç University, İstanbul, Türkiye

**Keywords:** Pyruvate, NMR assignment, Isotopic labeling

## Abstract

The use of *Escherichia coli* for recombinant protein production is a cornerstone in structural biology, particularly for nuclear magnetic resonance (NMR) spectroscopy studies. Understanding the metabolic behavior of *E. coli* under different carbon sources is critical for optimizing isotope labeling strategies, which are essential for protein structure determination by NMR. Recent advancements, such as mixed pyruvate labeling, have enabled improved backbone resonance assignment in large proteins, making selective isotopic labeling strategies more important than ever for NMR studies. In this study, we aimed to investigate the metabolic adaptations of *E. coli* when grown on pyruvate as the sole carbon source, a common condition used to achieve selective labeling for NMR spectroscopy. Using NMR-based metabolomics, we tracked key metabolic shifts throughout the culture process to better understand how pyruvate metabolism affects protein production and isotopic labeling. Our results reveal that pyruvate is rapidly depleted before IPTG induction, while acetate and lactate accumulate due to overflow metabolism. These byproducts persist after induction, indicating that pyruvate is diverted into waste pathways, which limits its efficient use in isotope incorporation. This metabolic inefficiency presents a challenge for isotopic labeling protocols that rely on pyruvate as a carbon source for NMR studies. Our results highlight the need to fine-tune pyruvate supplementation to improve metabolic efficiency and isotopic labeling, making this study directly relevant to optimizing protocols for NMR studies involving protein structure determination. These insights provide valuable guidance for enhancing the quality and yield of isotopically labeled proteins in NMR spectroscopy.

**Supplementary Information:**

The online version contains supplementary material available at 10.1007/s10858-025-00462-1.

## Introduction

Recombinant protein production is a crucial technique for conducting research on proteins. The desired protein can be produced in a laboratory setting using organisms such as *Escherichia coli* and, after purification, can be used in various studies. One of the methods used to determine the structures of the produced and purified proteins is nuclear magnetic resonance (NMR) spectroscopy (Wüthrich 1990). Since not all atomic nuclei are NMR-active, it is necessary to use specific isotopes in the sample. For a nucleus to be NMR-active, its spin must be greater than zero. Although some nuclei with integer spins are NMR-active, nuclei with half-integer spins, such as ^1^H, ^13^C, and ^15^N, produce the most useful signals due to their narrow absorption ranges (Gerothanassis et al. 2002). Isotopically labeled protein production follows a different path than standard recombinant protein production protocols. Minimal media are employed in isotopic labeling processes. Unlike rich media, minimal media do not contain amino acids. Instead, compounds containing elements such as nitrogen, carbon, sulfur, calcium, and sodium are added along with a carbon source (e.g., glucose) (Azatian et al. 2019). Any of the minimal medium components can be added as isotopically labeled, allowing to produce proteins with specific labeled atoms. The atom attached to the radical group is referred to as the alpha carbon, while the carbon belonging to the carboxyl group is called the beta carbon. The backbone of proteins consists of nitrogen from the amino group, the alpha carbon, and the beta carbon. When both carbons are NMR-active, the resonance of one atom affects the other, reducing the sensitivity of the measurement. This issue is resolved by producing proteins with ^13^C-labeled alpha carbons and ^12^C-labeled beta carbons (Robson et al. 2018). The use of labeled pyruvate as a carbon source, instead of labeled glucose, has been reported in the literature (Robson et al. 2018), enabling selective labeling of only the alpha carbons of amino acids. Some amino acids are synthesized directly from pyruvate, while others are produced through the gluconeogenesis pathway or the Krebs cycle. In all cases, the main carbon backbone of pyruvate is largely preserved, and the carbon atoms at specific positions are incorporated as alpha or beta carbons in the structure of amino acids. By using pyruvate labeled at the second or third carbon position, only the alpha carbons of amino acids can be selectively labeled, allowing for the collection of NMR data with higher sensitivity (Robson et al. 2018). Furthermore, this approach also simplifies resonance assignments.

While glucose-labeled protein production typically requires multiple NMR experiments to complete chemical shift assignments, our results demonstrate that using mixed pyruvate labeling enables these assignments to be accomplished with a single NMR dataset. However, a significant challenge in pyruvate-labeled protein production today lies in bacterial growth. Often, bacteria either fail to grow or grow very slowly and at low levels. While pyruvate-based isotopically labeled protein production is an effective method for resonance assignments, it also has some drawbacks. Compared to nutrient-rich media, the amount of protein produced is significantly lower, leading to a higher cost per unit of protein. The minimal medium, which contains only various salts, a single nitrogen source, and a single carbon source, slows down bacterial metabolism, reducing the amount of protein produced. When pyruvate is used as the carbon source, certain amino acids are synthesized through the reversal of the glycolysis pathway. Since glycolysis operates in reverse to synthesize amino acids, a pathway typically used for energy production, this reversal negatively impacts bacterial metabolism and slows down protein production. Additionally, isotopically labeled chemicals are expensive due to the high production costs. As a result of these combined factors, the use of minimal medium and pyruvate as the carbon source results in a disproportionately high cost relative to the amount of protein produced. Therefore, there is a need to improve the protein production process when using pyruvate as the carbon source. While pyruvate has been explored as an alternative carbon source for isotopic labeling in protein production, its impact on the overall metabolic transformations in bacterial culture medium remains under-investigated. Understanding how pyruvate influences metabolite composition and bacterial metabolism is critical for optimizing culture conditions and improving the efficiency of protein production. In this study, we aim to systematically investigate the metabolic changes in the bacterial culture medium when pyruvate is used as the sole carbon source. By analyzing the metabolic shifts and metabolite profiles in the medium, we provide new insights into how pyruvate drives bacterial metabolic pathways, which can have broader implications for biotechnological processes and isotope labeling strategies.

## Materials and methods

### Preparation of pyruvate-based M9 medium and bacterial growth

The GST fusion protein-tagged UbcH8 on the PGEX vector was transformed into *E. coli* BL21(DE3) cells and grown in LB medium with antibiotics at 37 °C for 15 h. A pyruvate-based minimal medium was prepared and sterilized as described by Robson et al. (2018). Bacteria were transferred from the LB medium to the minimal medium, resuspended, and grown at 37 °C for 6–8 h to adapt to the new conditions. Optical density was measured hourly to monitor growth. When the optical density at 595 and 600 nm reached 0.3, samples were taken for NMR analysis. The incubator temperature was reduced to 22 °C to promote proper protein folding (Rosano and Ceccarelli 2014). At an optical density of 0.6, IPTG was added to induce protein production, which continued for 15 h. All samples were centrifuged and stored at -80 °C for NMR analysis.

### NMR sample preparation

After thawing the frozen medium, 500 µL was taken, and 55 µL of a buffer solution prepared with deuterium oxide was added. The added solution contained 37.7 mM Na₂HPO₄, 12.3 mM KH₂PO₄, 20 mM NaCl, and 1 mM DSS. DSS was included as a reference for the NMR analyses and was added to achieve a final concentration of 0.1 mM. The prepared samples were placed in 5 mm NMR tubes, and measurements were performed. NMR data acquisition was carried out using a 500 MHz Bruker Ascend magnet, equipped with an Avance NEO console and a BBO double resonance room temperature probe. The 1D NOESY-presat (noesygppr1d) pulse sequence was applied for all NMR data collections, with each spectrum consisting of 8 K scans and 32 K complex data points, over a spectral width of 9615.4 Hz. Chenomx NMR Suite 9.02 software (Chenomx Inc., Canada) was utilized to calculate the concentrations of target metabolites (Dağ et al. 2022). Metabolite concentrations and the peak shapes of other compounds were determined by Chenomx, based on the concentration and peak height of DSS. NMR data were collected with 8 K scans to enhance the signal-to-noise ratio.

To analyze the NMR-based metabolomics data, MetaboAnalyst 6.0, an online platform designed for comprehensive metabolomics data processing and statistical analysis, was utilized. Prior to statistical evaluations, the dataset was pre-processed using cube root transformation and Pareto scaling to normalize the data and reduce the influence of large variations among metabolites. Partial Least Squares Discriminant Analysis (PLS-DA) was employed as a supervised method to further discriminate between the time points and highlight key metabolites driving the separation. PLS-DA, a powerful statistical tool, maximized the separation between predefined groups, in this case, different time points during the growth and production phases. The Variable Importance in Projection (VIP) scores obtained from PLS-DA identified the most influential metabolites contributing to the temporal metabolic changes observed in the bacterial culture.

## Results and discussion

### Metabolite profiling in the culture medium

**Fig. 1:**
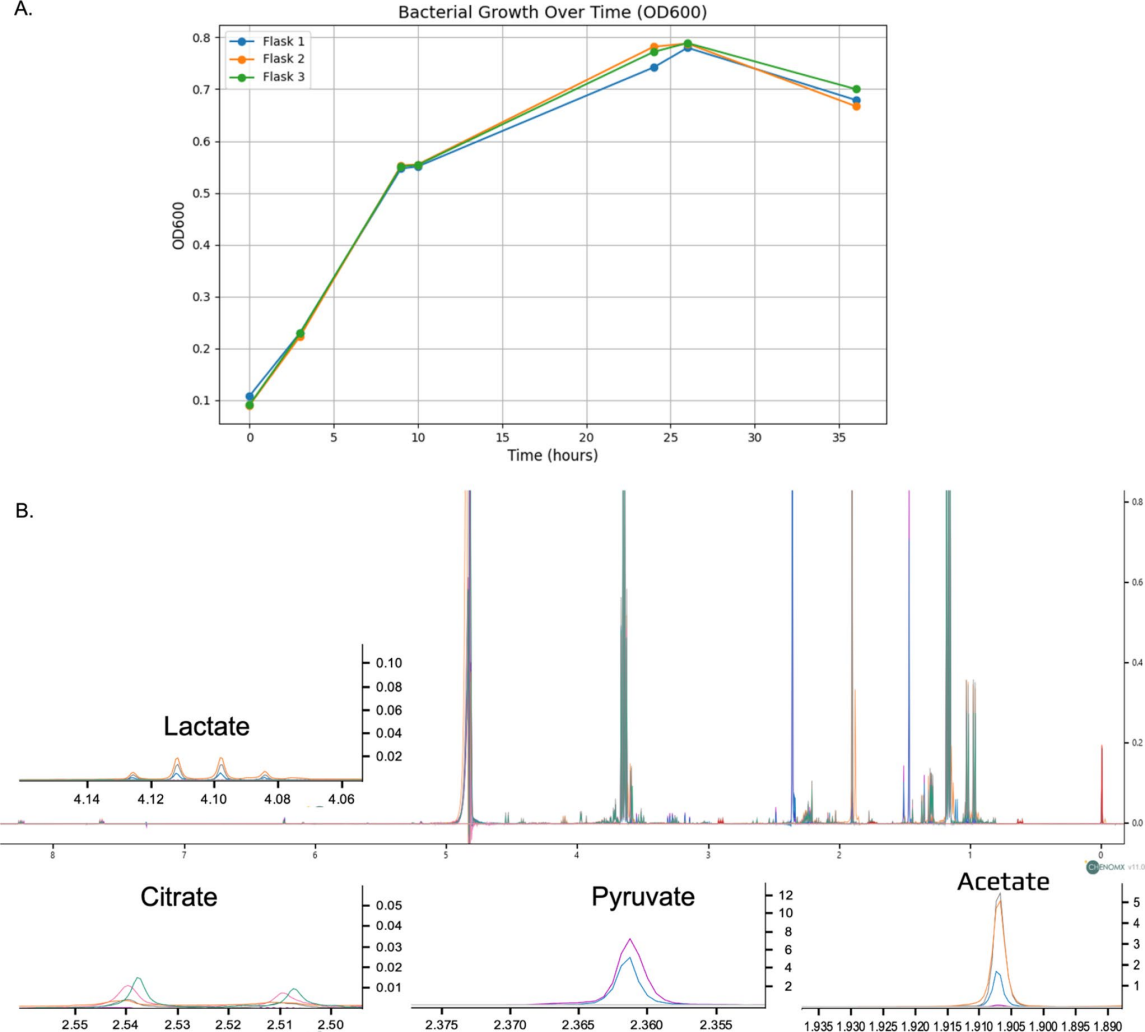
**(A)** Comparative Analysis of Bacterial Growth in Three Independent Cultures Over 36 h, Monitored by Optical Density at 600 nm (B) **B** Overlay of full NMR spectra at different production stages, with detailed views of key metabolites: lactate, citrate, pyruvate, and acetate, illustrating their concentration dynamics throughout the production process. (0 h (purple), 3 h (blue), 9 h (orange), 10 h (gray), 24 h (pink), 26 h (green)

The optical density (OD) measurements at 600 nm were taken at various time points to assess the bacterial growth in three separate flasks (Fig. 1A). By 24 h (14 h post-induction), the cultures reached their peak OD readings, with Flask 1 at 0.742, Flask 2 at 0.782, and Flask 3 at 0.772. Figure 1B displays the full NMR spectra overlaid across different production stages, illustrating the temporal dynamics of metabolites in the bacterial culture. Key metabolites such as lactate, citrate, pyruvate, and acetate are highlighted with expanded views to provide a more detailed analysis.

**Fig. 2:**
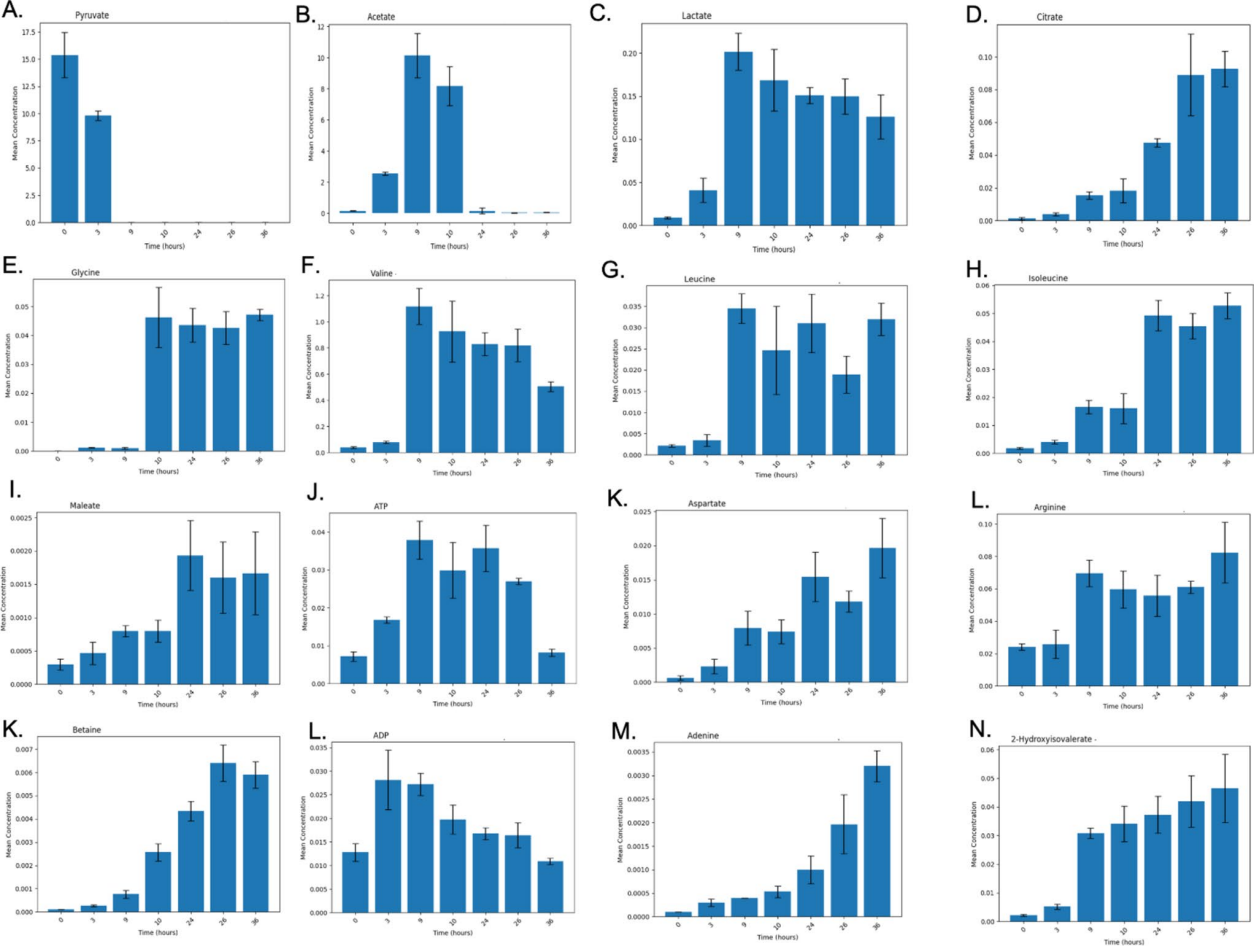
Time-Dependent Concentration Profiles of Key Metabolites during Bacterial Growth and Recombinant Protein Production. **A**. Pyruvate, **B**. Acetate, **C**. Lactate, **D**. Citrate, **E**. Glycine, **F**. Valine, **G**. Leucine, **H**. Isoleucine, **I**. Maleate, **J** ATP, **K**. Aspartate, **L**. Arginine, **M**. Adenine, **N**. 2-Hydroxyisovalerate

The bar plots in Fig. 2 illustrate the temporal concentration changes of key metabolites involved in central metabolic pathways during bacterial growth and recombinant protein production. These metabolites were measured at various time points: 0 h, 3 h, 9 h (IPTG induction), 10 h, 24 h, 26 h, and 36 h, capturing metabolic dynamics from the lag phase through the exponential and stationary phases of bacterial growth. The analysis of metabolite concentrations across different time points revealed significant changes in key intermediates involved in central carbon metabolism during bacterial growth and recombinant protein production. These metabolic shifts were observed both before and after IPTG induction, reflecting the cells’ metabolic response to the high availability of pyruvate as the primary carbon source.

Pyruvate (Fig. 2A), the main carbon source in this experiment, exhibited a rapid decrease starting well before IPTG induction, with its levels already substantially reduced by the 9-hour mark. At 9 h, just prior to the addition of IPTG for protein induction, the OD values had further increased to approximately 0.55 in all flasks. Early decline in pyruvate suggests that bacterial cells are actively consuming pyruvate through multiple metabolic pathways to support energy production and biosynthesis. The sustained reduction of pyruvate post-9 h further indicates that the cells continue to use pyruvate intensively during recombinant protein production. The early depletion of pyruvate is likely a result of its diversion into multiple pathways, including both the TCA cycle for energy production and overflow metabolic pathways. Concurrent with the depletion of pyruvate, both acetate (Fig. 2B) and lactate (Fig. 2C) showed substantial increases in the culture media prior to IPTG induction. This observation suggests that bacterial cells, faced with an abundance of pyruvate, are converting excess pyruvate into acetate and lactate as a means of mitigating the buildup of intracellular pyruvate. These metabolites are likely excreted into the medium as waste products to avoid toxic intracellular accumulation. The high concentrations of acetate and lactate in the culture media reflect overflow metabolism, a process whereby excess carbon from glycolysis (in the form of pyruvate) is diverted into pathways that produce acetate and lactate, which are then secreted from the cells. This process is typical of bacterial cultures experiencing rapid growth and high glycolytic flux, where the TCA cycle becomes saturated and alternative metabolic routes are used to handle the surplus carbon.

After IPTG induction at 9 h, the concentrations of acetate and lactate in the medium remained elevated. The sustained high levels of acetate and lactate post-IPTG likely reflect the fact that once these metabolites are expelled into the culture medium, their reuptake and reintegration into intracellular metabolism is limited. Acetate and lactate, once secreted, may no longer serve as useful substrates for energy production or biosynthesis. Instead, their accumulation in the medium suggests that the bacterial cells are unable, or inefficient, at reclaiming these byproducts. Citrate (Fig. 2D), a key intermediate of the TCA cycle, showed a gradual increase in concentration, particularly after IPTG induction. This suggests that while pyruvate is being converted into acetate and lactate, a portion of it continues to fuel the TCA cycle to meet the energy demands and biosynthetic needs of the cells. Citrate’s rise is indicative of active TCA cycle flux, particularly after recombinant protein production begins, as the cells require sustained energy and carbon skeletons for the synthesis of amino acids and other macromolecules. Together, the patterns observed in pyruvate, acetate, lactate, and citrate concentrations highlight the metabolic flexibility of bacterial cells in response to pyruvate availability. Before IPTG induction, excess pyruvate is channeled into overflow pathways, resulting in the secretion of acetate and lactate into the medium. Following IPTG induction, this pattern continues, but the rise in citrate suggests that a portion of the pyruvate is also directed into the TCA cycle to support the increased metabolic demands of recombinant protein production. The secretion of acetate and lactate into the culture media throughout the experiment underscores the cells’ need to expel excess metabolites and maintain intracellular metabolic balance under conditions of high pyruvate flux.

Glycine (Fig. 2E) shows stable concentrations before IPTG induction, followed by a modest increase during the later time points. Branched-chain amino acids (BCAAs), including valine (Fig. 2F), leucine (Fig. 2G), and isoleucine (Fig. 2H), show moderate increases post-IPTG induction, indicative of their essential roles in protein synthesis. The upregulation of BCAA biosynthesis following IPTG induction reflects the heightened demand for amino acids during recombinant protein production. The concentration of maleate (Fig. 2I) increases gradually, peaking at 24 h, suggesting its involvement in energy production pathways as bacterial cells transition into the stationary phase. ATP (Fig. 2J) levels rise significantly after IPTG induction, peaking at 10 h, indicating an increased demand for energy during the early stages of recombinant protein synthesis. This is followed by a gradual decline in ATP levels, reflecting a stabilization of energy metabolism as the bacterial culture approaches stationary phase. The ADP (Fig. 2L) profile mirrors that of ATP, peaking at 10 h and gradually decreasing, indicating high energy turnover during protein production.

Aspartate (Fig. 2K) and arginine (Fig. 2L) show continuous increases in concentration after IPTG induction, with aspartate peaking at 24 h. This suggests their critical roles in supporting protein synthesis and nitrogen metabolism, particularly as amino acid demand rises during recombinant protein production. Similarly, betaine (Fig. 2K) concentrations increase over time, peaking at 24 h. Betaine is known to function as an osmoprotectant, and its accumulation may reflect bacterial stress responses during extended periods of growth and protein synthesis. Adenine (Fig. 2M), a purine nucleotide, increases steadily following IPTG induction, peaking at 36 h, indicating heightened nucleotide turnover associated with increased DNA/RNA synthesis during recombinant protein production. Finally, 2-hydroxyisovalerate (Fig. 2N), a metabolite linked to branched-chain amino acid metabolism, shows a gradual increase over time, peaking at 36 h. This suggests its involvement in secondary metabolic pathways, particularly as bacterial cells shift toward the stationary phase and metabolic processes become focused on maintenance rather than growth.

Taken together, these results reveal a clear metabolic reprogramming in response to IPTG induction and recombinant protein production. Pyruvate consumption, acetate overflow, and shifts in amino acid and nucleotide metabolism reflect the bacterial cells’ need to balance energy production with the synthesis of macromolecules for protein production. The most significant metabolic changes occur between 9 and 10 h, shortly after IPTG induction, indicating the critical metabolic adjustments required to meet the demands of protein synthesis. By 24 h (14 h post-induction), the cultures reached their peak OD readings, with Flask 1 at 0.742, Flask 2 at 0.782, and Flask 3 at 0.772. At 26 h (16 h post-induction), the OD values remained stable around 0.78 for all flasks, indicating continued growth. However, at the 36-hour time point (26 h post-induction), a slight decrease in OD was observed across all flasks, with values dropping to 0.679 in Flask 1, 0.667 in Flask 2, and 0.700 in Flask 3. This decline suggests that the bacterial cultures may have entered the stationary phase or experienced nutrient depletion in the medium. As the bacterial culture transitions to the stationary phase (24–36 h), metabolic activity stabilize s, as evidenced by the gradual leveling off of key metabolite concentrations.

### Multivariate analysis of metabolite profiles

**Fig. 3:**
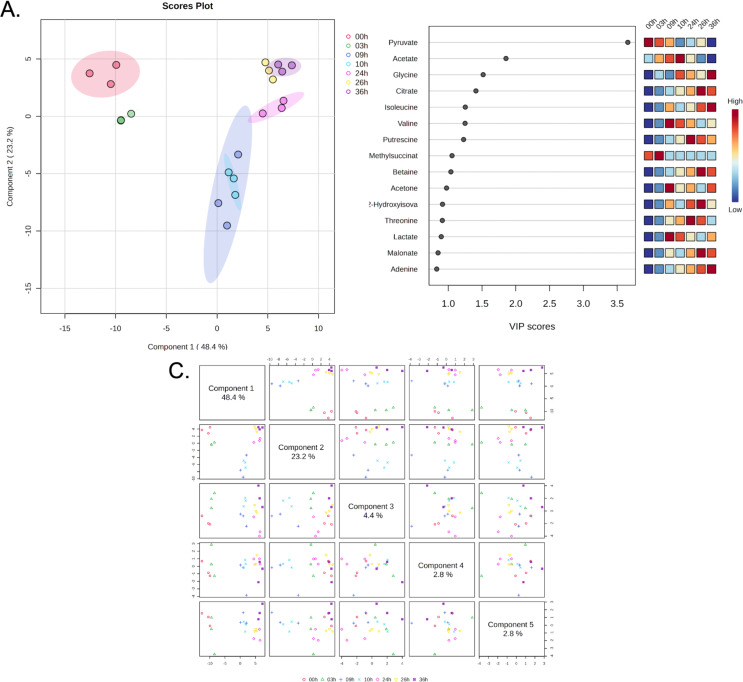
Multivariate Analysis of Metabolite Profiles at Different Time Points. **(A)** Partial Least Squares Discriminant Analysis (PLS-DA) Scores Plot highlighting the discriminative power of PLS-DA, with Component 1 explaining 48.4% of the variance and Component 2 explaining 22.3%. The plot shows distinct separation of metabolic profiles over time, similar to the PLS-DA results. **(B)** Pairwise Plot of PLS-DA Components showing the relationship between components generated by the PLS-DA model. **(C)** Variable Importance in Projection (VIP) Scores from the PLS-DA model, ranking metabolites based on their contribution to class separation. Metabolites such as acetate, glycine, and citrate are the top contributors to the separation between time points, with corresponding heat maps showing their concentration changes over time

Partial Least Squares Discriminant Analysis (PLS-DA) was performed to further explore the metabolic changes in response to IPTG induction and identify metabolites that contribute most to the observed variations across time points. The PLS-DA scores plot (Fig. 3A) highlights the discriminative power of the PLS-DA model, which maximizes the separation between metabolite profiles at different time points. Component 1 and Component 2 in the PLS-DA model are the first two latent variables that maximize the separation between predefined groups. In this plot, Component 1 explains 48.4% of the variance, and Component 2 explains 22.3%, together capturing a significant portion of the total variability. The clear separation of the time points along these two components reflects the dynamic changes in bacterial metabolism over time, particularly in response to IPTG induction at the 9-hour mark. Pre-induction time points, 0 h, 3 h, and 9 h, form distinct clusters, with 9 h showing more separation from earlier time points, as expected, given that IPTG induction occurs at this time. Interestingly, the 9 h and 10 h samples (1 h post-IPTG induction) show a sharp metabolic shift, as these two clusters are well separated, indicating a rapid metabolic reprogramming following IPTG addition. This shift is likely due to the metabolic demands imposed by recombinant protein production, which alters the cellular utilization of pyruvate and other metabolites involved in central carbon metabolism and amino acid biosynthesis. The pairwise scatter plots of the first five components of the PLS-DA model (Fig. 3B) provide a more detailed breakdown of how each component contributes to the separation of time points. As shown in the plot, Component 1(48.4%) and Component 2 (22.3%) account for most of the variance, with clear separation of early (0 h, 3 h) and post-induction time points. This further supports the observation that IPTG induction introduces significant metabolic shifts, which are well captured by the first two components. The pairwise component plots confirm that the metabolic changes induced by IPTG are most strongly captured by Components 1 and 2, with minimal variance explained by subsequent components. This suggests that the major metabolic reprogramming in response to IPTG induction can be largely described by the shifts along these two axes, with smaller components reflecting minor metabolic adjustments as the cultures reach the stationary phase.The PLS-DA analysis provides a supervised view of the temporal metabolic shifts in bacterial cultures during recombinant protein production. The analysis confirms that IPTG induction at 9 h is a major driver of metabolic variation, leading to rapid and distinct metabolic changes as bacteria adapt to the demands of protein synthesis. The stabilization of metabolite profiles at later time points, as seen in both the scores plot and pairwise component plots, reflects the transition into the stationary phase, where nutrient consumption slows, and the metabolic state becomes more consistent.

To further understand the metabolites driving the separation of time points in the PLS-DA analysis, we calculated the Variable Importance in Projection (VIP) scores (Fig. 3C). VIP scores rank metabolites based on their contribution to the PLS-DA model, identifying those most influential in distinguishing between time points, particularly in response to IPTG induction and the subsequent phases of bacterial growth and protein production.

The metabolites with the highest VIP scores are displayed in Fig. 3C, indicating their importance in explaining the metabolic shifts across the time points. The top-ranking metabolites include pyruvate, acetate, glycine, citrate, isoleucine, and valine, which are directly or indirectly involved in energy metabolism, amino acid biosynthesis, and central carbon metabolism. These metabolites are particularly important in the context of bacterial growth under minimal medium conditions, where pyruvate serves as the sole carbon source.

Its importance in the VIP score suggests that shifts in glycine metabolism are closely linked to the demands of protein production, especially after IPTG induction, when the need for amino acids increases. The VIP score analysis identifies key metabolites that are critical in explaining the metabolic shifts observed during the bacterial growth phases and in response to IPTG induction. Pyruvate, acetate, glycine, citrate, and branched-chain amino acids play pivotal roles in supporting energy metabolism and protein biosynthesis under minimal medium conditions with pyruvate as the carbon source.

These findings provide a comprehensive overview of how central metabolism is reprogrammed in response to the metabolic demands imposed by recombinant protein synthesis (Table 1).

**Table 1:**
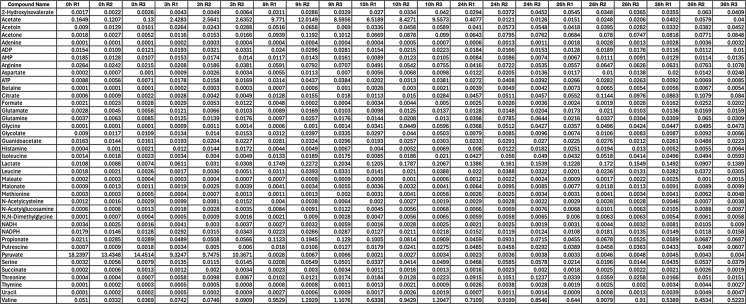
Concentration of metabolites at different time points during bacterial growth and recombinant protein production. The concentrations of selected metabolites measured in the culture media at multiple time points (0h, 3h, 9h, 10h, 24h, 26h, and 36h). Metabolite concentrations are reported for three biological replicates (R1, R2, and R3) for each time point

## Discussion and conclusions

The metabolic analysis provides significant insights into the adaptations of *E. coli* when grown with pyruvate as the sole carbon source. Our results highlight the complex balance between central carbon metabolism and overflow pathways under both basal conditions and recombinant protein production.

### Pyruvate utilization and overflow metabolism

In bacterial cells, the regulation of intracellular metabolite levels is essential for maintaining metabolic balance and cellular homeostasis. Excess accumulation of specific metabolites can disrupt cellular processes and impede efficient metabolism. To avoid such disruptions, bacteria employ various mechanisms to expel unnecessary or excessive metabolites from the cell. For instance, waste products generated during metabolic processes, such as lactate, ethanol, or acetate, are often transported out of the cell to prevent intracellular buildup. Additionally, when certain metabolites, such as amino acids or organic acids, accumulate in large amounts, they can be exported to restore metabolic equilibrium. This export is mediated by specialized transporter proteins located in the bacterial membrane, which facilitate the active or passive transport of metabolites out of the cell. Efflux pumps, commonly associated with the removal of toxic compounds and antibiotics, also play a role in expelling surplus or unwanted metabolites. These transport and efflux systems are vital for bacterial survival, as they help preserve cellular homeostasis, optimize metabolic efficiency, and mitigate the toxic effects of accumulated byproducts. Understanding these mechanisms is critical for insights into bacterial metabolism and potential applications in biotechnology and medicine. Pyruvate, as expected, was rapidly consumed by the bacterial cells, with its concentration significantly reduced well before IPTG induction. This early depletion of pyruvate suggests its immediate utilization in key metabolic pathways such as glycolysis, the citric acid (TCA) cycle, and amino acid biosynthesis. The pivotal role of pyruvate in *E. coli* metabolism is well established, serving not only as an energy source but also as a precursor for various biosynthetic reactions that are critical for cell growth and maintenance (Zhang et al. 2019). Our data are consistent with previous studies that have demonstrated the versatile role of pyruvate in driving both energy production and biosynthesis during the exponential phase of bacterial growth (Zhu et al. 2020). Pyruvate-based labeling has garnered significant interest for its ability to selectively label specific positions in amino acids, thereby simplifying chemical shift assignments and improving sensitivity in NMR spectroscopy (Robson et al. 2018). However, the metabolic challenges associated with using pyruvate as the sole carbon source, including the accumulation of overflow metabolites and slower bacterial growth, limit its widespread application. Recent studies, such as Chae et al. (2012), have highlighted the importance of tracing metabolic changes during recombinant protein production to better understand the stress responses and metabolic adjustments in *E. coli*. Their analysis of metabolite profiles along the time course of protein expression revealed significant fluctuations in amino acid and nucleotide levels, emphasizing the metabolic burden associated with recombinant protein synthesis. In our study, we expand on these findings by systematically comparing the metabolic dynamics of *E. coli* under pyruvate-based and glucose-based labeling conditions. Our results show that pyruvate consumption leads to the accumulation of acetate and lactate as overflow metabolites, while glucose labeling typically exhibits a different metabolic profile, characterized by higher glycolytic flux. These insights, combined with the detailed metabolite profiling presented in Chae et al. (2012), underscore the need for optimization strategies to balance growth efficiency with isotopic labeling requirements.

Prior to IPTG induction, we observed significant accumulation of both acetate and lactate in the culture medium, indicating that excess pyruvate was being diverted into overflow metabolic pathways. This phenomenon, where surplus pyruvate is converted to byproducts such as acetate and lactate, is well documented in rapidly growing bacterial cultures, particularly when carbon availability exceeds the oxidative capacity of the TCA cycle (Holms 1986). The accumulation of these metabolites in the extracellular environment suggests that *E. coli* cells are expelling them to prevent intracellular metabolic imbalances. Once expelled, the re-uptake and subsequent metabolism of acetate and lactate appear to be inefficient, which could explain why their concentrations remain elevated throughout the experiment. This observation is in line with studies reporting that the re-assimilation of acetate and lactate by *E. coli* is energetically costly and tightly regulated (Vemuri et al. 2006; Wolfe 2005). The inability of the cells to efficiently reclaim acetate and lactate likely reflects a strategy to maintain metabolic homeostasis by eliminating surplus carbon. Once pyruvate is converted to these byproducts and secreted, their re-utilization may be energetically unfavorable, particularly under conditions where the TCA cycle is already operating at full capacity. This highlights the overflow metabolism phenomenon, which is exacerbated when pyruvate availability exceeds the oxidative needs of the cells (Basan et al. 2015). The persistent high levels of acetate and lactate in the medium further suggest that the cells prioritize maintaining intracellular balance over attempting to reclaim and metabolize these byproducts, thus allowing their excretion into the extracellular environment (Enjalbert et al. 2015).

### Metabolic shifts Post-IPTG induction

Following IPTG induction at 9 h, we observed that the concentrations of acetate and lactate remained elevated. This continued presence of overflow metabolites suggests that the metabolic burden imposed by recombinant protein production does not alleviate overflow metabolism, but rather intensifies it. During high metabolic demands, such as during recombinant protein synthesis, the increased carbon flux through glycolysis and the TCA cycle may further limit the cells’ ability to fully oxidize pyruvate, leading to continued diversion into acetate and lactate pathways (El-Mansi et al. 2006). Citrate, a key intermediate of the TCA cycle, is essential for energy generation and the production of biosynthetic precursors. Its accumulation suggests that the cells are attempting to balance energy production with the increased demand for biosynthetic precursors necessary for protein synthesis (Luo et al. 2020). This finding is consistent with earlier work demonstrating that, under recombinant protein production conditions, bacterial cells often increase TCA cycle activity to meet the elevated demand for ATP and precursor metabolites (Gill et al. 2000). Despite this increased TCA cycle flux, the persistent high levels of acetate and lactate indicate that the cells are unable to fully process the available pyruvate through oxidative pathways, leading to its continuous conversion into overflow metabolites. This metabolic response likely represents a physiological adaptation to the stress imposed by recombinant protein production, where the diversion of pyruvate into overflow pathways helps to prevent metabolic overload within the cell (Basan et al. 2015).

### Implications for bioprocess optimization

The results of this study have several important implications for optimizing bacterial growth conditions in biotechnological applications. The accumulation of acetate and lactate can have deleterious effects on bacterial growth and protein production, as these byproducts contribute to acidification of the culture medium and inhibit key cellular processes (Enjalbert et al. 2015). Strategies to mitigate the accumulation of these byproducts, such as optimizing pyruvate feeding rates or engineering strains with improved acetate and lactate assimilation capabilities, could enhance the efficiency of recombinant protein production (Vemuri et al. 2006; Sauer et al. 2011). Additionally, the findings underscore the importance of fine-tuning carbon fluxes in metabolic pathways to minimize the diversion of carbon into overflow metabolites. Engineering bacterial strains with an enhanced capacity for pyruvate oxidation, or increasing the TCA cycle’s capacity to process higher fluxes of carbon, could help reduce the reliance on overflow pathways, thereby improving growth and protein production yields (Holms 1986). Further investigation into strain engineering strategies that enhance carbon utilization while reducing byproduct formation could prove beneficial for large-scale industrial fermentation processes (Sauer et al. 2011).

Pyruvate has been widely explored as a carbon source for isotopic labeling due to its ability to selectively label specific positions in amino acids, providing improved sensitivity and simplified resonance assignments for NMR spectroscopy (Robson et al. 2018). Recent advancements in this field have focused on optimizing *E. coli* host strains and culture conditions to enhance the efficiency of pyruvate-based labeling. For instance, Kelpšas et al. (2021) demonstrated the evolution of *E. coli* strains for efficient deuterium labeling of recombinant proteins using sodium pyruvate-d3, highlighting the potential of modified host strains to overcome challenges such as reduced growth rates and metabolic inefficiencies. This study provides critical insights into the metabolic behavior of *E. coli* during recombinant protein production when grown on pyruvate as the sole carbon source. The rapid depletion of pyruvate, coupled with the significant accumulation of acetate and lactate in the medium, underscores the challenges associated with pyruvate over-supplementation. Excess pyruvate, while serving as a critical substrate for energy production and biosynthesis, is inefficiently utilized under these conditions, leading to its diversion into overflow pathways and the excretion of byproducts. These findings highlight the importance of fine-tuning carbon source supplementation in biotechnological processes, as supplying pyruvate in excessive amounts can lead to metabolic imbalances and the inefficient use of carbon resources. By optimizing the levels of pyruvate provided and improving the metabolic capacity of the cells to process it, the accumulation of waste byproducts like acetate and lactate can be minimized, ultimately enhancing the efficiency of recombinant protein production. Future work should focus on strain engineering and process optimization to better manage carbon fluxes, reduce waste, and improve the overall yield and productivity of industrial fermentation systems. Rather than adding the full amount of pyruvate at the start of the culture, a titration-based approach, where pyruvate is supplemented in smaller amounts over time, could mitigate rapid depletion and overflow metabolism. Such an approach may enhance bacterial growth and protein production.

## Electronic supplementary material

Below is the link to the electronic supplementary material.

Supplementary Material 1

## Data Availability

The datasets generated during and/or analyzed during the current study are available from the corresponding author on reasonable request.
