# Supplementary material for: Exploring the biochemical landscape of bacterial medium with pyruvate as the exclusive carbon source for NMR studies
Source: J Biomol NMR. 2025 Mar 6;79(3):143–53. doi: 10.1007/s10858-025-00462-1 (PMC12378656; doi:10.1007/s10858-025-00462-1)
Supplement: Supplementary file 1 — Supplementary Material 1 [file 10858_2025_462_MOESM1_ESM.pdf]

Supplementary Methods: For unsupervised exploration of the dataset, Principal Component Analysis (PCA) was applied to reduce the dimensionality of the data while capturing the maximum variance between samples. PCA allowed visualization of the overall metabolic changes across different time points, revealing distinct clusters associated with the progression of bacterial growth and recombinant protein production, particularly in response to IPTG induction. Pearson correlation analysis was performed to assess the relationships between individual metabolites, followed by hierarchical clustering to group metabolites based on their correlation patterns. These analyses revealed distinct clusters of metabolites that are co-regulated, providing insights into the metabolic pathways modulated during recombinant protein production.

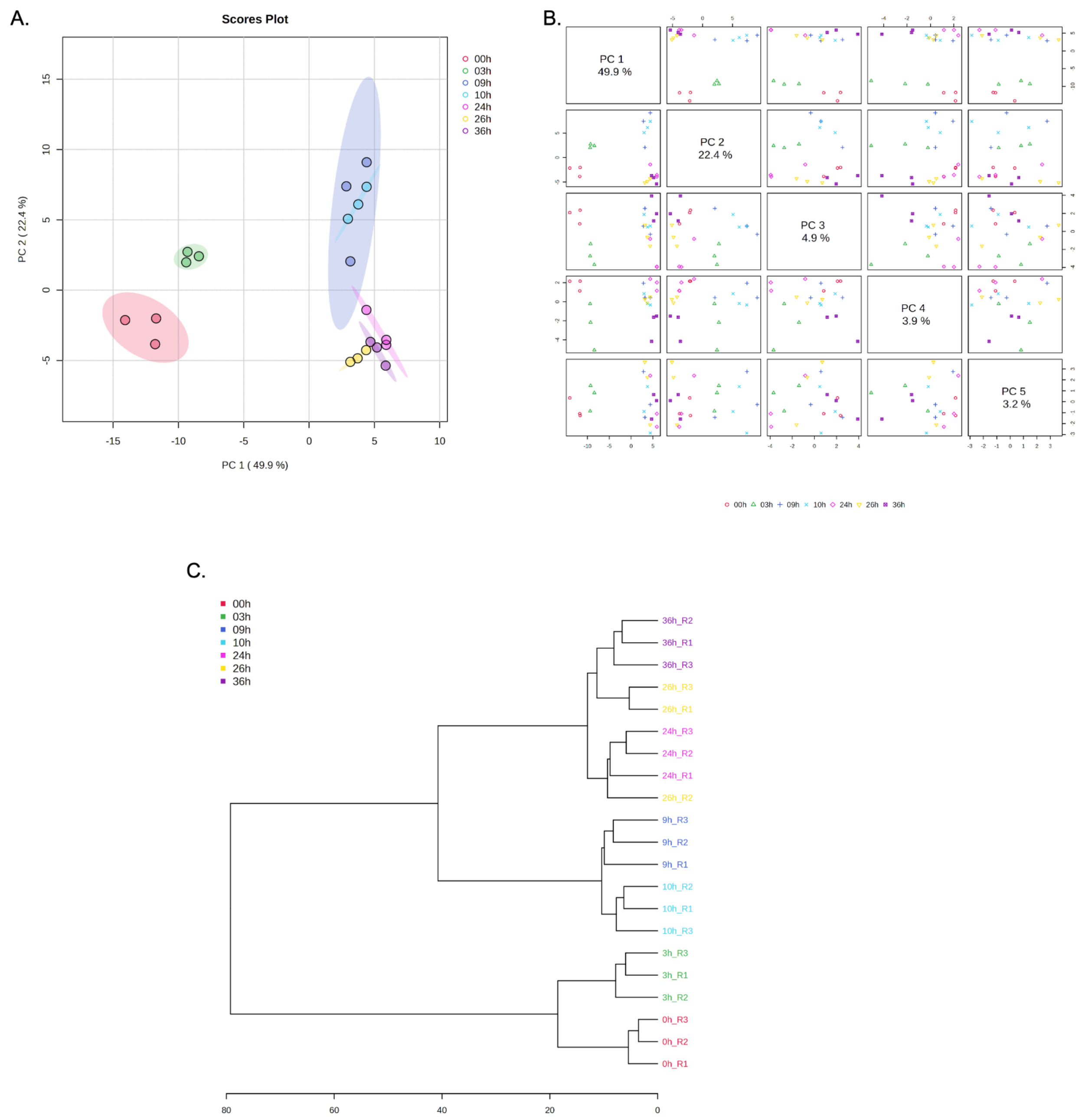

Supplementary Figure 1. A. Principal Component Analysis (PCA) Scores Plot showing the separation of metabolite profiles at various time points (0h, 3h, 9h, 10h, 24h, 26h, and 36h). The PCA demonstrates distinct clustering of samples from different time points, with PC1 explaining 49.9% of the variance and PC2 explaining 22.4%. **B. Pairwise Plot of Principal Components (PCs)**, illustrating the relationships between different PCs from the PCA analysis. **C.**

Hierarchical Clustering Dendrogram visualizing the similarity of metabolite profiles across replicates at different time points, indicating clear time-dependent grouping of samples.

The PCA scores plot (Supp. Figure 1A) highlights distinct separation of metabolite profiles across time points, with the first two principal components (PC1 and PC2) explaining 49.9% and 22.4% of the total variance, respectively. PC1 and PC2 represent the first and second principal components in the PCA, which capture the largest variance in the dataset. The samples collected at 0h and 3h cluster tightly, indicating minimal metabolic variation during the early growth (lag) phase when bacterial metabolism is primarily focused on adjusting to the medium. However, a significant shift occurs between 3h and 9h, corresponding to the addition of IPTG at the 9-hour mark to induce recombinant protein production.

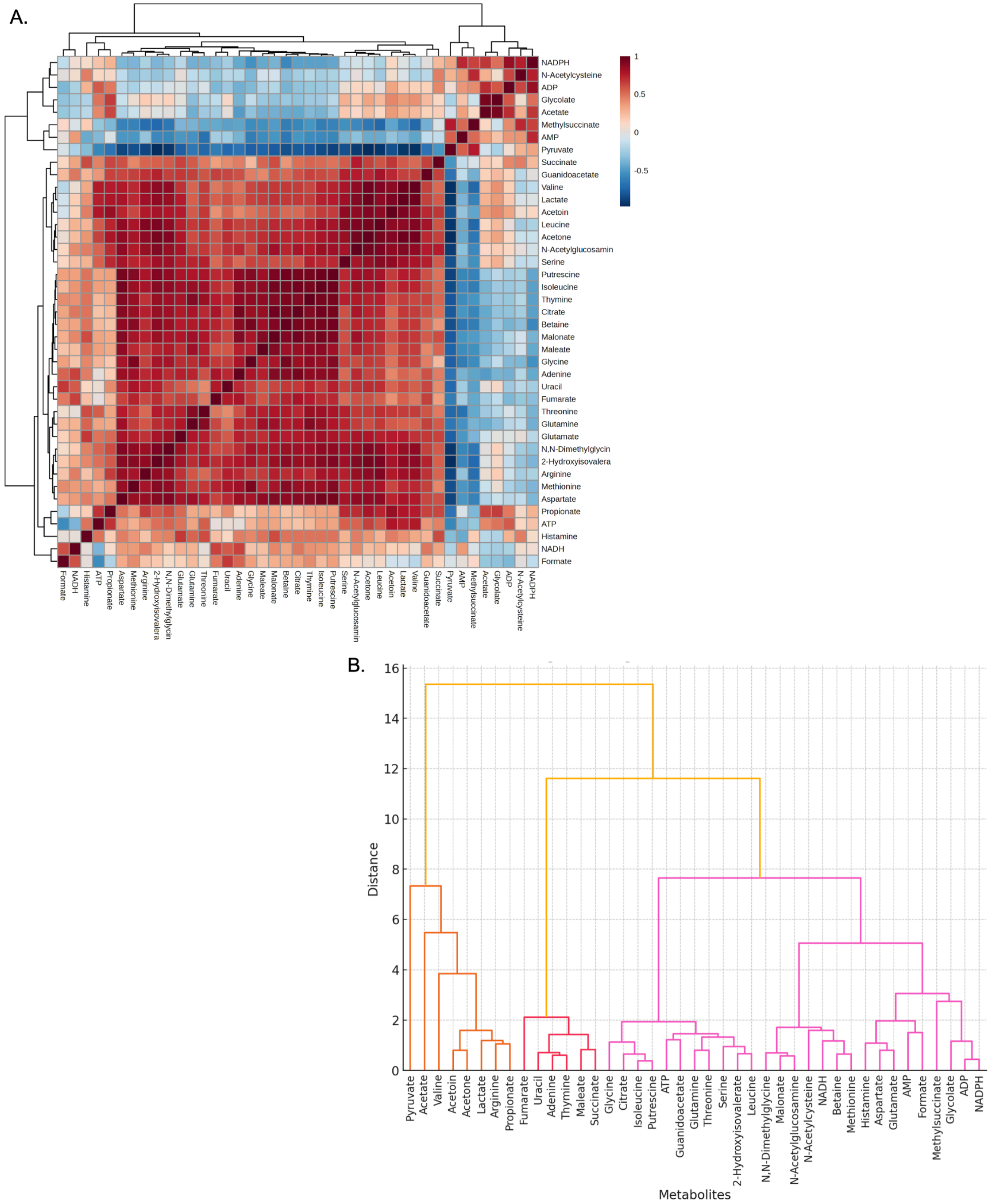

Supplementary Figure 2. Correlation Matrix and Hierarchical Clustering of Metabolites in Bacterial Cultures during Recombinant Protein Production

A. Heatmap of Pearson Correlation Coefficients between metabolites, illustrating the pairwise correlations of metabolite levels during different phases of bacterial growth and protein production. The color scale ranges from strong positive correlations (red) to strong negative correlations (blue). Metabolites involved in energy metabolism, amino acid biosynthesis, and central carbon metabolism form distinct clusters, reflecting their co-regulation during bacterial growth. **B. Hierarchical Clustering Dendrogram** showing the relationships between metabolites based on similarity in their correlation patterns. Metabolites are grouped into clusters based on their correlation distances, with closer distances indicating higher similarity in their metabolic roles. Key metabolites such as pyruvate, acetate, and branched-chain amino acids (BCAAs) are highlighted as major contributors to metabolic shifts during the recombinant protein production process.

Supplementary information about supplementary figures

Post-induction, the 10h samples (1 hour after IPTG induction) show a distinct shift along PC1, indicating rapid metabolic changes following protein induction. This is likely due to the metabolic burden of recombinant protein synthesis, which alters the demand for energy and metabolic intermediates derived from pyruvate. Notably, the 24h and 26h time points (14 and 16 hours after IPTG induction, respectively) form closely clustered groups, suggesting that the bacteria have reached a more stable metabolic state during this extended period of protein production. This clustering is consistent with the bacteria entering the stationary phase, during which growth slows and metabolism stabilizes, with a potential shift toward metabolite recycling and waste product excretion (e.g., acetate, lactate).

By 36h (26 hours post-induction), the metabolite profile has shifted slightly compared to earlier post-IPTG time points, likely due to nutrient depletion or accumulation of metabolic byproducts in the culture medium. The confidence ellipses show tight clustering of replicate samples at later time points (24h, 26h, 36h), indicating reproducibility of metabolic states during and after recombinant protein production. The PCA results clearly indicate that IPTG induction at 9h causes a dramatic metabolic shift, with the most pronounced changes occurring between 9h and 10h. These shifts reflect the metabolic adaptations required for the bacteria to support increased protein synthesis, including changes in energy metabolism, amino acid synthesis, and metabolite excretion. The pairwise scatter plots of principal components (Supp. Figure 1B) provide a more detailed view of the variance captured by the top five components. The first two principal components (PC1 and PC2) capture most of the variance, as indicated by the clear clustering of samples according to time points, especially around IPTG induction (9h-10h). Time points immediately following IPTG induction, such as 10h, show clear separation from pre-induction time points, illustrating the significant metabolic impact of initiating recombinant protein synthesis. Minor components, such as PC3 (4.9%) and PC4 (3.9%), capture additional subtle metabolic changes that may reflect variations in the levels of specific metabolites involved in later phases of growth and protein production. For instance, these components might be influenced by the accumulation of waste products like acetate or lactate as bacterial growth slows. In summary, the PCA analysis reveals significant metabolic shifts corresponding to the addition of IPTG at 9h, with marked differences in metabolite profiles between pre- and post-induction phases. The clustering of time points from 24h to 36h suggests that bacterial metabolism stabilizes during the later stages of protein production, possibly due to nutrient limitation or the plateauing of recombinant protein synthesis. To further investigate the relationships between the metabolite profiles at different time points during recombinant protein production, a hierarchical clustering analysis was performed (Supp. Figure 1C). The clustering

dendrogram groups samples based on their metabolic similarity, revealing clear temporal patterns in the data. The samples collected at 0h, 3h, and 9h (prior to or at the time of IPTG induction) form a distinct cluster, indicating that the metabolic profiles during the early phases of growth (lag and early exponential phases) are highly similar. The proximity of these time points suggests minimal metabolic reprogramming in the absence of IPTG and during the initial phase of bacterial growth in the pyruvate-supplemented medium. The 9h time point represents the moment IPTG is added to induce recombinant protein production, yet it remains metabolically similar to earlier time points, reflecting a delay in the metabolic response to IPTG. In contrast, the samples collected 1 hour after IPTG induction (10h) form a separate, highly distinct cluster from the earlier time points. This separation underscores the significant metabolic changes that occur rapidly following IPTG induction, likely driven by the metabolic demands associated with recombinant protein synthesis. These changes may include shifts in energy metabolism, amino acid biosynthesis, and central carbon metabolism, all necessary to support the increased biosynthetic activity. The 24h, 26h, and 36h time points (14, 16, and 26 hours post-IPTG induction, respectively) form a tight, well-defined cluster. This clustering indicates that once the bacteria have adapted to the protein production process, their metabolism stabilizes. The minimal differences between these time points suggest that bacterial cells have reached a stationary phase, with metabolic activities largely focused on maintaining cellular homeostasis and recycling metabolites. This stabilization is likely due to nutrient depletion and the plateauing of protein synthesis as bacterial growth slows down. The close grouping of replicates within these time points further demonstrates the reproducibility of the metabolic changes observed.

Interestingly, hierarchical clustering also reveals that some of the 9h replicates show partial overlap with 10h replicates, suggesting a transitional phase in metabolic reprogramming as cells switch from regular growth to recombinant protein production. This gradual transition is

expected, as the induction of recombinant protein synthesis imposes a substantial metabolic burden, requiring time for bacterial cells to adjust their metabolic pathways to meet the demands of protein synthesis. Overall, the hierarchical clustering analysis provides a clear temporal overview of the metabolic shifts that occur during bacterial growth and recombinant protein production. The clustering of time points prior to IPTG induction, followed by distinct metabolic states post-induction, highlights the significant metabolic reprogramming that accompanies protein production. This analysis confirms that bacterial metabolism stabilizes during the later stages of growth, likely in response to nutrient limitation and the completion of protein synthesis.

To understand the relationships between metabolites during recombinant protein production, we performed a Pearson correlation analysis, followed by hierarchical clustering to group metabolites based on their co-regulation patterns. These analyses provide insights into how metabolites interact with one another during different phases of bacterial growth. The heatmap in Supp. Figure 2A shows the Pearson correlation coefficients between metabolites across all time points, providing a comprehensive view of metabolite interactions. The color scale ranges from dark red (strong positive correlation, close to +1) to dark blue (strong negative correlation, close to -1), with white indicating no correlation. Several distinct clusters of highly correlated metabolites emerge, suggesting that these groups are co-regulated during bacterial growth and recombinant protein production. Pyruvate, a central metabolite in glycolysis and the citric acid cycle, shows strong positive correlations with several key energy-related metabolites such as acetate, succinate, and citrate, reflecting its pivotal role in energy metabolism and carbon flow through these pathways. The tight clustering of these metabolites suggests that they are part of a coordinated metabolic response to the use of pyruvate as the sole carbon source. In contrast, metabolites such as NADPH and NADH, which are involved in redox reactions and cellular energy balance, display negative correlations with certain amino acids like isoleucine and valine,

possibly reflecting a metabolic trade-off between energy production and biosynthesis during recombinant protein production. This is consistent with the metabolic burden that bacteria face when diverting resources toward protein synthesis. The metabolites involved in amino acid metabolism, such as leucine, valine, and isoleucine (branched-chain amino acids, BCAAs), form a highly correlated cluster. These BCAAs play critical roles in protein synthesis and are tightly regulated during recombinant protein production, particularly after IPTG induction. Their strong correlations with other amino acids, such as serine and glycine, suggest that the demand for amino acids during the growth and production phases drives coordinated metabolic shifts.

Additionally, several metabolites related to amino acid biosynthesis and protein production, such as isoleucine, valine, and citrate, form another cluster. The clustering of these metabolites suggests that they are closely linked to the metabolic demands of recombinant protein synthesis, especially following IPTG induction when the demand for amino acids sharply increases. This cluster also includes succinyl-CoA, an intermediate in the TCA cycle, further indicating the importance of energy production and carbon flow through central metabolism to support protein biosynthesis.

The hierarchical clustering dendrogram (Supp. Figure 4B) further elucidates the relationships between metabolites based on their correlation profiles. The dendrogram groups metabolites into clusters according to their similarity in correlation patterns, with the vertical axis representing the distance (or dissimilarity) between clusters. Metabolites with similar roles or functions are grouped together, indicating shared regulatory mechanisms or metabolic pathways. At the top of the dendrogram, pyruvate and acetate form a distinct cluster, reflecting their central roles in energy metabolism and overflow metabolism, respectively. The strong correlation between pyruvate and acetate likely reflects the bacterial metabolism's response to the high flux of carbon through glycolysis and the citric acid cycle, leading to acetate secretion as a byproduct during

later stages of growth. Interestingly, metabolites involved in nucleotide metabolism, such as adenine and uracil, cluster together, reflecting their role in DNA and RNA synthesis, which may be essential for supporting bacterial growth and protein production during the exponential phase. This cluster also includes metabolites like thymine, emphasizing the coordinated regulation of nucleotide metabolism in response to the energy and biosynthetic demands imposed by recombinant protein production. Overall, the hierarchical clustering reveals that metabolite profiles are organized into functional groups based on their roles in central carbon metabolism, amino acid biosynthesis, and energy production. The tight clustering of key metabolites involved in pyruvate metabolism and BCAA biosynthesis highlights the importance of these pathways in supporting bacterial growth and protein production under minimal medium conditions with pyruvate as the sole carbon source. The Pearson correlation heatmap and hierarchical clustering dendrogram together reveal distinct metabolic modules that are co-regulated during bacterial growth and recombinant protein production. Metabolites involved in energy metabolism, amino acid biosynthesis, and nucleotide metabolism show strong correlations, reflecting the bacterial cells' need to balance energy production with biosynthesis to support protein production.
